# Explainable artificial intelligence in air traffic control: effects of expertise on workload, acceptance, and usage intentions

**DOI:** 10.1186/s40708-025-00287-6

**Published:** 2026-01-24

**Authors:** Giulia Cartocci, Alexandre Veyrié, Nicola Cavagnetto, Christophe Hurter, Augustin Degas, Ana Ferreira, Mobyen Uddin Ahmed, Shahina Begum, Shaibal Barua, Bianca Maria Serena Inguscio, Vincenzo Ronca, Gianluca Borghini, Gianluca Di Flumeri, Fabio Babiloni, Pietro Aricò

**Affiliations:** 1 Departmental Faculty of Medicine and Surgery, Saint Camillus University of Health Sciences, 00131 Rome, Italy; 2 BrainSigns Srl, Industrial Neurosciences Lab, 00198 Rome, Italy; 3 Fédération ENAC ISAE-SUPAERO ONERA, Université de Toulouse, 31400 Toulouse, France; 4 Deepblue Srl, 00185 Rome, Italy; 5 Artificial Intelligence and Intelligent Systems Group, Mälardalen University, Västerås, 72220 Sweden; 6 Department of Computer, Control, and Management Engineering, Sapienza University of Rome, 00185 Rome, Italy; 7 Department of Physiology and Pharmacology, Sapienza University of Rome, 00185 Rome, Italy; 8 College of Computer Science and Technology, Hangzhou Dianzi University, Hangzhou, 310005 China

**Keywords:** Explainable artificial intelligence, Air traffic control, Electroencephalography, Workload, Expertise

## Abstract

Explainability is crucial for establishing user trust in Artificial Intelligence (AI), particularly within safety-critical domains such as Air Traffic Management (ATM) and Air Traffic Control (ATC). This study empirically investigates the effects of Explainable AI (XAI), specifically HeatMap-based visual explanations, on cognitive workload, user acceptance, and intention to use AI-driven decision-support systems among Air Traffic Control Officers (ATCOs). Despite significant theoretical advancements in the broader XAI domain, empirical evidence addressing the specific impact of visual explanations on human-AI interactions in safety-critical environments like ATC remains limited. To address these critical gaps, an experimental comparison was conducted between explainable (HeatMap) and non-explainable (BlackBox) AI conditions, involving two user groups: expert and student ATCOs. Both objective neurophysiological measures (Electroencephalography) and subjective questionnaires were employed to capture comprehensive user responses. Key findings revealed that the presence of visual explanations significantly reduced cognitive workload and enhanced users’ willingness to adopt the AI system, regardless of participants’ level of expertise. However, explicit perceptions of AI’s impact on work performance were predominantly influenced by expertise, with less experienced controllers reporting a greater perceived impact than their expert counterparts. By combining objective neurometrics with subjective user assessments, this research advances methodological rigor in evaluating human-AI interactions and highlights the importance of tailored, user-centric explanations. These findings directly contribute to practical guidelines for designing cognitively compatible and trustworthy AI tools in ATC, providing nuanced insights for targeted training and deployment strategies based on user expertise.

## Introduction

Artificial intelligence (AI) has become a pivotal component in modern Air Traffic Management (ATM) and Air Traffic Control (ATC), driven by the growing complexity and volume of global air traffic. Traditional systems, reliant on human controllers for real-time decision-making, are increasingly augmented by AI algorithms that support predictive analytics, conflict detection, and dynamic routing [1–4]. These systems process massive streams of radar, weather, and flight data far faster and more accurately than human operators can, enabling earlier detection of potential conflicts and more efficient use of airspace. As a result, Air Traffic Control Officers (ATCOs), whose main duty is to prevent the collision of airplanes, are shifting from direct tactical control toward supervisory roles, where they oversee and intervene in the actions of automated systems [5–8]. This transition introduces new cognitive demands, requiring controllers to maintain situational awareness in the presence of opaque or rapidly changing AI-driven decisions [9–11]. Integration challenges remain, especially in balancing human judgment with algorithmic recommendations, but the trend toward collaborative human-AI control paradigms is reshaping the operational and cognitive landscape of ATC [12–15].

The integration of AI in critical decision-making environments, such as ATC, necessitates ensuring that AI-driven recommendations are both transparent and comprehensible to human operators. Transparency is particularly crucial in these safety-critical fields, where understanding the rationale behind AI-generated solutions can directly affect human trust, acceptance, and operational performance [16–18]. This requirement is emphasized by regulatory authorities such as the European Union Aviation Safety Agency (EASA) who establishes that: “A crucial component of achieving Trustworthy AI is transparency which encompasses three elements: traceability, explainability and open communication about the limitations of the AI system”. Consequently, AI systems must deliver more than just technical performance; they must also offer strong explainability. Furthermore, EASA defines explainability as the “capability to provide the human with understandable, reliable, and relevant information with the appropriate level of detail and with appropriate timing on how an AI application produces its results”.

Explainable Artificial Intelligence (XAI), encompassing methods that clarify AI decision-making processes, has thus emerged as an essential research domain, addressing concerns around interpretability, reliability, and trustworthiness of AI systems [19–26]. In a recent and comprehensive survey of the emerging field of XAI, aiming to address the opacity of complex AI systems, Arrieta et al., (2020) proposed a unified, user-centered definition of explainability and presented two structured taxonomies: one for general machine learning models and another specifically for deep learning approaches. They highlighted key challenges in achieving effective XAI, including the trade-off between interpretability and model performance, the absence of standard evaluation metrics, and the need for context-aware, user-adapted explanations [20]. In another recent review, Degas et al., (2022) identified two critical gaps in the current landscape of XAI for ATM. On one hand, they highlighted the urgent need for a shift toward user-centric XAI, where systems are not only technically capable but also designed to align with the needs, workflows, and mental models of ATCOs [21]. Much of the existing research remains system-focused, prioritizing what AI can do, rather than what users actually need to understand [16, 25].

Explanations are often chosen arbitrarily by researchers, ranging from global overviews (e.g., full decision trees) to local snippets (e.g., individual decision branches), with little input from end users. On the other hand, they carried out attention to a persistent confusion between the terms transparency and explainability, a conceptual ambiguity that continues to hinder progress in XAI. Although the difference may seem nuanced, clarity is essential. Transparency refers to system-level openness (e.g., exposing internal mechanics), while explainability focuses on user-facing clarity and relevance (e.g., offering contextualized, understandable insights) [27]. In this way, a tool can be transparent yet not explainable, such as revealing a neural network’s architecture without making its outputs interpretable to an ATCO, or explainable without being transparent, as in summarizing a system’s decision while hiding proprietary algorithms. These concepts, though related, are not interchangeable; both are crucial to fostering trust in AI.

In this paper, we draw a clear distinction between transparency and explainability, as these terms are often blurred in related work. Transparency refers to an inherent property of a system, whereby its internal functioning can be directly inspected and understood without additional mediation (e.g., rule-based systems or decision trees). Explainability, by contrast, denotes post-hoc methods or interfaces that provide users with accessible reasons or visualizations of the system’s outputs when the underlying model is not inherently transparent (e.g., saliency maps, heatmaps, or surrogate models). The present study focuses on explainability, since the AI model employed for conflict detection and resolution is not transparent by design but requires additional explanatory interfaces to make its recommendations interpretable to ATCOs.

Despite significant theoretical advances in the broader domain of XAI, empirical evidence regarding the effectiveness of specific visual explanation methods, such as HeatMap-based visualizations, remains scarce within the aviation sector. Given the highly dynamic and cognitively demanding nature of ATC tasks [28–31], understanding how such visual explanations influence cognitive workload, user acceptance, and intention to use AI-driven tools is paramount. Prior studies indicate that visual explanatory methods can serve as cognitive anchors, facilitating rapid comprehension and efficient decision-making [32, 33], although their effectiveness may depend significantly on the user’s level of expertise and pre-existing operational routines [34–36]. In support of this view, [22] recently presented a framework for integrating XAI into ATC, highlighting a crucial shift in the design of XAI systems for ATCOs. Rather than assuming that explanations are universally beneficial, the authors argue that explanations should be tailored to the specific instances when and why ATCOs actually need them. Their study, grounded in operational goals of ATM, found that explanations are particularly valuable when ATCOs must document decisions or resolve conflicts between their judgment and AI-generated advisories. However, when the AI system aligns with the controller’s expectations, explanations are often unnecessary and may even contribute to cognitive overload. This user-centered approach emphasizes that effective XAI in ATM must be context-aware, delivering relevant explanations only when they enhance trust, transparency, or accountability in human-AI collaboration [26]. These findings underscore the importance of moving beyond system-level design to examine how explanatory interfaces concretely affect ATCOs’ cognitive experience and behavioral intentions, particularly in terms of mental workload, system acceptance, and willingness to adopt AI support tools in practice.

Mental workload, user acceptance, and intention to adopt AI-driven systems are interconnected human factors critical to the successful integration of AI in ATC. Mental workload, defined as the cognitive resources required by a human operator to perform specific tasks, directly impacts both performance and safety in high-stakes domains such as aviation [29, 37]. Mental workload is a central consideration in the adoption of AI-based decision support systems in ATC, as the integration of automation can both alleviate and amplify cognitive demands depending on task complexity, system transparency, and user expertise [28–30]. Excessive cognitive load can lead to decreased situational awareness, delayed reaction times, and increased error rates, underscoring the necessity of designing AI support tools that effectively mitigate, rather than exacerbate, cognitive demands on ATCOs [9, 31, 38]. Consequently, AI systems must be designed not only for technical reliability but also for cognitive compatibility with human operators.

This has direct implications for system acceptance and intention to use, two key dimensions in technology adoption frameworks such as the Technology Acceptance Model (TAM) [39, 40]. Furthermore, the acceptance of AI-driven decision aids among ATCOs hinges significantly on the perceived utility, ease of use, and reliability of the system [34, 41]. Trust in AI systems, shaped by transparency and explainability, fundamentally affects user acceptance and determines whether controllers incorporate AI recommendations into their operational routines or dismiss them entirely [17, 21]. Lastly, intention to use AI tools is strongly predicted by these acceptance factors and the controller’s subjective evaluation of the system’s effectiveness in supporting their operational tasks. Research consistently highlights that ATCOs’ intention to adopt AI systems increases when explanations provided by the AI align with their cognitive expectations, operational experiences, and professional judgment [22, 33].

Hence, addressing these dimensions collectively, mental workload, acceptance, and usage intention, is vital for fostering effective human-AI teaming in the safety-critical environment of ATC. Research shows that when AI tools are perceived as supportive, particularly when their outputs are transparent and cognitively aligned with operator needs, they are more likely to be accepted and incorporated into routine ATC workflows [8, 27]. Conversely, if AI decisions are perceived as opaque or cognitively burdensome, operators may disengage, resist adoption, or default to manual control, particularly in high-stakes situations [42]. In this context, explainability serves as a mediating factor between system complexity and user acceptance by reducing cognitive uncertainty and enhancing perceived usefulness [20, 25]. However, explainability itself must be carefully designed; overly complex or redundant explanations may increase workload rather than mitigate it [35]. Thus, the relationship between AI explainability, cognitive workload, and intention to use is highly dynamic, requiring empirical validation within real-world operational settings such as ATC.

Electroencephalography (EEG) and derived neurometrics, quantitative indicators extracted from brain activity signals that reflect cognitive states such as mental workload, have emerged as reliable and objective tools for assessing mental effort across various contexts, enabling empirical validation of human-AI interactions under ecologically valid conditions [28, 43]. Nonetheless, studies on the differences in cognitive workload and user acceptance that may occur between experienced and inexperienced ATCO under different task difficulties are quite insufficient in the literature. Addressing these knowledge gaps, the present study empirically investigates how visual explanations, specifically HeatMap-based XAI, influence cognitive workload, user acceptance, and willingness to adopt AI support tools among ATCOs. A combination of EEG-based neurometric assessments and targeted subjective questionnaires was used in the present study to capture both implicit and explicit user responses. Acknowledging the critical role of expertise, the current experimental design contrasts expert ATCOs instructors against less experienced student controllers, thereby enabling an in-depth exploration of how expertise modulates responses to visual AI explanations. In this way, this research work seeks not only to extend existing theoretical frameworks by empirically validating the impact of visual explanations on human factors in AI-supported ATC systems but also to address critical gaps identified by prior literature reviews and expert recommendations related to XAI systems in ATC [20, 25, 34].

Based on the literature, three research questions have been formulated to address key gaps identified in the previous sections:

- RQ1: To what extent does XAI (HeatMap-based visualization) influence the cognitive workload of ATCOs compared to a non-explainable (black-box) AI system?
- RQ2: How does the provision of XAI affect user acceptance and intention to use AI decision-support tools in ATC?
- RQ3: Does the expertise level of ATCOs moderate the effects of XAI on workload, acceptance, and intention to use?

From these research questions, some hypotheses can be directly investigated in the present work:

**H1** *XAI (visual explanations using HeatMap) reduces cognitive workload (measured via EEG neurometrics) compared to a non-explainable (black-box condition) AI system.*

**H2** *XAI increases users’ intention to use the AI tool compared to a non-explainable AI system (BlackBox).*

**H3** *Acceptance of AI tools (measured via EEG neurometrics and subjective measures) is higher among student ATCOs than expert ATCOs.*

**H4** *The impact of XAI on intention to use and acceptance depends significantly on the user’s expertise.*

## Materials and methods

### Participants

In total, 21 participants were recruited at the École Nationale de l’Aviation Civile (ENAC, Toulouse, France), the first European training school for ATCOs and pilots, to participate in the study. Participants were divided into two groups based on expertise: “Expert” and “Student” air traffic controllers (Table 1).

The Expert group included 11 participants (3 female; mean age: 41 years, range 34–51). This group was mostly composed of ENAC ATCO instructors (seven), with additional participants being former ATCOs now working in research (two) or in training/formation roles (two). On average, the experts had 15 years of operational experience, primarily in en-route control, though several also had approach and tower backgrounds.

The Student group included 10 participants (4 female; mean age: 22 years, range 20–26). These were ENAC trainees enrolled in the final stage of their formation, just before being assigned to an operational control centre. This ensured that, while not yet licensed, they had completed nearly all academic and simulator training and were able to perform conflict-resolution tasks in a realistic experimental setting.

All participants were healthy volunteers with no history of neurological or psychiatric disorders, and with normal or corrected-to-normal vision. Exclusion criteria included refusal or inability to provide informed consent or the presence of medical conditions incompatible with EEG recording. The study was approved by the Research Ethics Committee of Toulouse University and conducted in accordance with the Helsinki Declaration and institutional requirements. All participants provided written informed consent prior to participation and were free to withdraw at any time.

**Table 1:** Summary of experimental participants involved in the study

Participants	Description
Expert group (Professional ATCOs)	Total of 11 professional ATCOs
	3 female (27%) and 8 male (73%)
	Mean age of 41 years (ranging between 34–51 years old)
	Mean 15 years of working experience
	Operational background: Only 2 participants did not have experience in En route ACC, but both had Approach ACC and Tower ATC experience
Student group	Total of 10 student ATCOs
	4 female (40%) and 6 male (60%)
	Mean age of 22 years (ranging between 20–26 years old)

### Experimental task

One of the principal functions of ATC is to ensure that aircraft do not conflict, that is, do not approach each other sufficiently close to give rise to the possibility of collision. In this study, a Conflict Detection & Resolution Task [44, 45] was performed using a Genetic Algorithm [46]. Genetic Algorithms (GA) are metaheuristic-based algorithms inspired by the process of natural selection that belong to the larger class of evolutionary algorithms and are commonly used to generate high-quality solutions to optimization and search problems by relying on biologically inspired operators such as mutation, crossover and selection [47–50]. As a population and evolutionary-based metaheuristic, the Genetic Algorithm (GA) means that a GA tries to iteratively improve candidate solutions according to some predefined criteria. In the case of conflict resolution, a candidate solution for the GA is a set of modified or not trajectories. Candidate solutions forming the population are evaluated in function of three criteria: the duration of the conflicts, if any; the length of the trajectories; and the number of changes of direction (i.e. order that must be given to implement this candidate solution). Once the GA has evaluated all candidate solutions in the population, it selects a set of candidate solutions, mostly the best, but also other candidate solutions to better explore the solution space. The algorithm then applies a set of mutation and crossover operations in an attempt to enhance the population and to possibly converge toward one of the optimal solutions [46, 49].

**Fig. 1:**
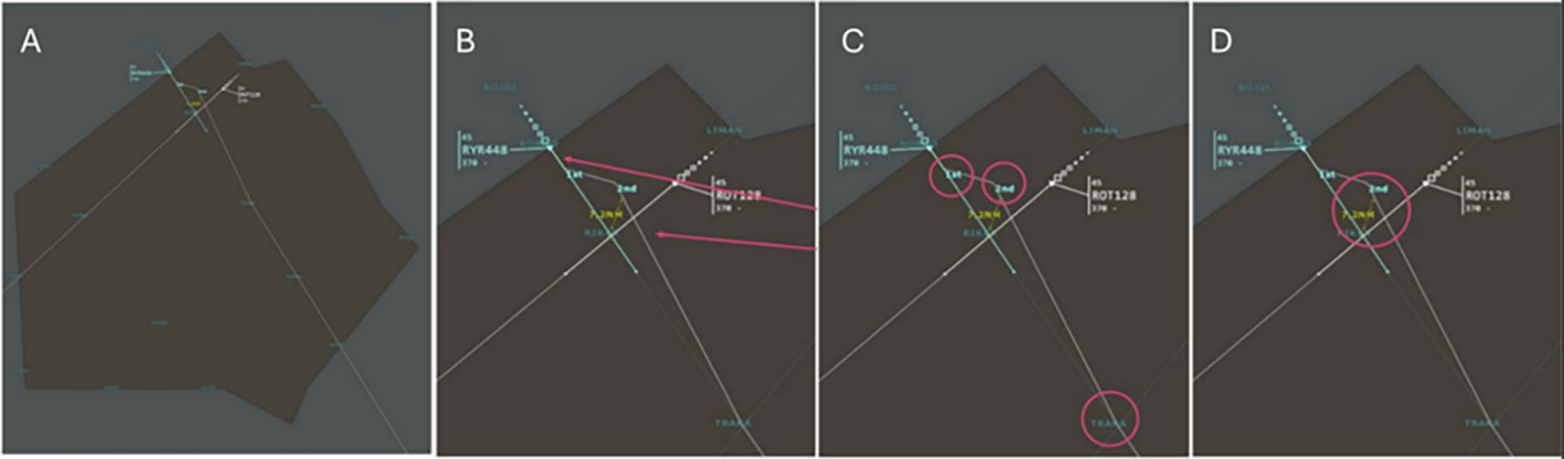
In the BlackBox (BB) explanation, the subject is presented with the trajectory of the aircraft and any trajectory changes (**A**). When the trajectory of an aircraft is modified, the initial trajectory of the aircraft appears in colour (in this case, cyan) and the modified trajectory appears in white (**B**). Each aircraft and its course changes have a unique associated colour. In this example, the trajectory of flight RYR448 is modified, while the trajectory of the ROT128 aircraft remains unchanged. The RYR448 aeroplane must first modify its trajectory at point 1 (First), then at point 2 (Second). It continues its trajectory after reaching the TRAKA point (**C**). Finally, the minimum distance between the two aeroplanes will be shown in yellow (in this case, 7.2 Nautical Meters). When the distance is less than 7 NM, it is indicated in orange, and the associated trajectories are displayed in yellow-green (**D**)

To sum up, in the Conflict Detection and Resolution task, the GA explores the possible solutions for a given conflicting situation between aircraft and extracts one solution which can be qualified as the “best” one with the given optimization criteria (e.g., number of actions, length of the trajectory, and number of orders). In order to provide explanations for the proposed solution, two different types of data presentation were used as follows:

BlackBox (BB): Simple visualization which only displays the proposed solution by the GA, enhanced by instructions to proceed (Fig. 1). Aeroplane trajectories are coloured differently. The minimal distance between aeroplanes is computed and displayed in yellow. The control orders that must be given by the ATCO to the different aeroplanes are placed along the trajectory, as well as their ordering (First, Second,... ).

This data presentation is not an explanation by itself, but the simple data presentation of the “best” solution the GA managed to extract. Compared to the existing system, the BlackBox data representation directly provides a solution to a detected conflict, while the system currently used only displays the detected conflicting aircraft without further information to solve it.

**Fig. 2:**
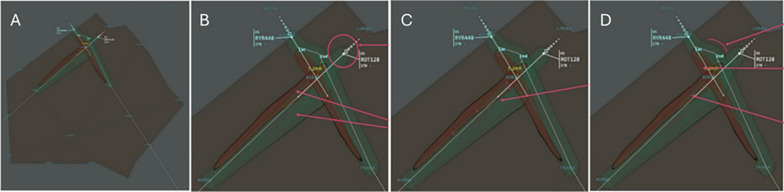
In the HeatMap (HM) explanation, the algorithm tests different trajectories and detects any associated conflicts (**A**). The HeatMap shows to the subject for each aircraft both information: i) an envelope of “good modifications” of trajectory (in green, >7NM) and ii) an envelope of “bad modifications” of trajectory (in red, < 7NM). Thus, the HeatMap the subject sees is a representation of the accumulation of the number of trajectories explored in the same place (**B**). To identify the plane corresponding to the green envelope, the subject can refer to the starting point of the zone and the current location of the plane. The red envelope follows the path of the associated green envelope (**C**). Finally, when two zones (green/red) overlap, this means that the plane can pass through this zone (in this case, ROT128) provided that the second plane (RYR448) changes its trajectory sufficiently (**D**)

Heatmap (HM): To make the rationale behind the proposed solution more explicit, the presentation highlighted both the “best” solution identified by the GA and the broader solution space explored, including both favorable and unfavorable trajectories. Heatmaps of the explored trajectories were displayed to show how aircraft paths could be safely modified (Fig. 2).

For example, the operator could see that AFR3218 could either maintain its current trajectory or turn left (although this would be less efficient), while KLM1258 and EZY208 could not continue on their original trajectories and were required to turn left as the only safe option. In addition, the timing of possible trajectory changes was conveyed by the extent of the “safe zone” (green area) before it transitioned into a “dangerous zone” (red area). This visualization was generated from a cumulative density map of explored candidate solutions, where each trajectory was convolved with a Gaussian kernel and then accumulated. Such a technique helps visually define areas, also known as contour maps [51], and supports reasoning about both “good” and “bad” solutions.

The Heatmap was specifically designed to create an uncluttered view of the possible trajectory modifications, filtering out less relevant candidate solutions while emphasizing those that help controllers understand why a given solution was proposed and why not others. For instance, it enables contrastive questions such as: “Can IBE752 turn left instead of continuing straight, despite the AI suggesting a right turn?” (it should not), or “What happens if I extend the modification of the IBERIA trajectory for a longer period of time?” (it would not provoke any conflict with FIN2655).

Heatmaps are also widely used in Explainable AI (XAI), for example in techniques such as LIME, LRP, and SHAP, to illustrate the contribution of input features to model outputs [52–54]. In the ATC domain, this approach was chosen because it provides controllers with an intuitive visual anchor that highlights both safe and unsafe envelopes while aligning with the strong visual culture of ATC operations. Unlike textual explanations, which risk monopolizing attention and disrupting situational awareness in fast-paced environments, visual explanations such as heatmaps can be integrated directly into radar-like displays, offering cognitively efficient and operationally relevant support.

### Experimental setup

**Fig. 3:**
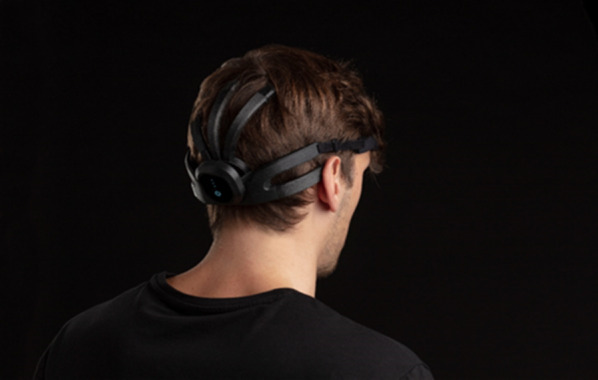
Mindtooth EEG headset, employed during the experiments

Scalp EEG was recorded with the wearable Mindtooth EEG headset (Fig. 3, Brain Products GmbH, Germany^1^) consisting of 8 electrodes positioned by following the International 10-20 EEG System (AFz, AF3, AF4, AF7, AF8, Pz, P3, P4), referenced to the right mastoid and grounded to the left one. The wet electrodes of the Mindtooth headset consist of open-cell, hydrophilic and highly absorbent cylindrical sponges. They harden when dry and become soft and expand when wet. The porous material is designed to absorb aqueous electrolyte solutions (1-2 % sodium chloride solutions are used as electrolytes). The electrolyte solution facilitates the establishment of an easy electrical connection to the participant’s skin. The impedance value was reduced to 100 k$\Omega$ before the start of the recording [55, 56].

**Fig. 4:**
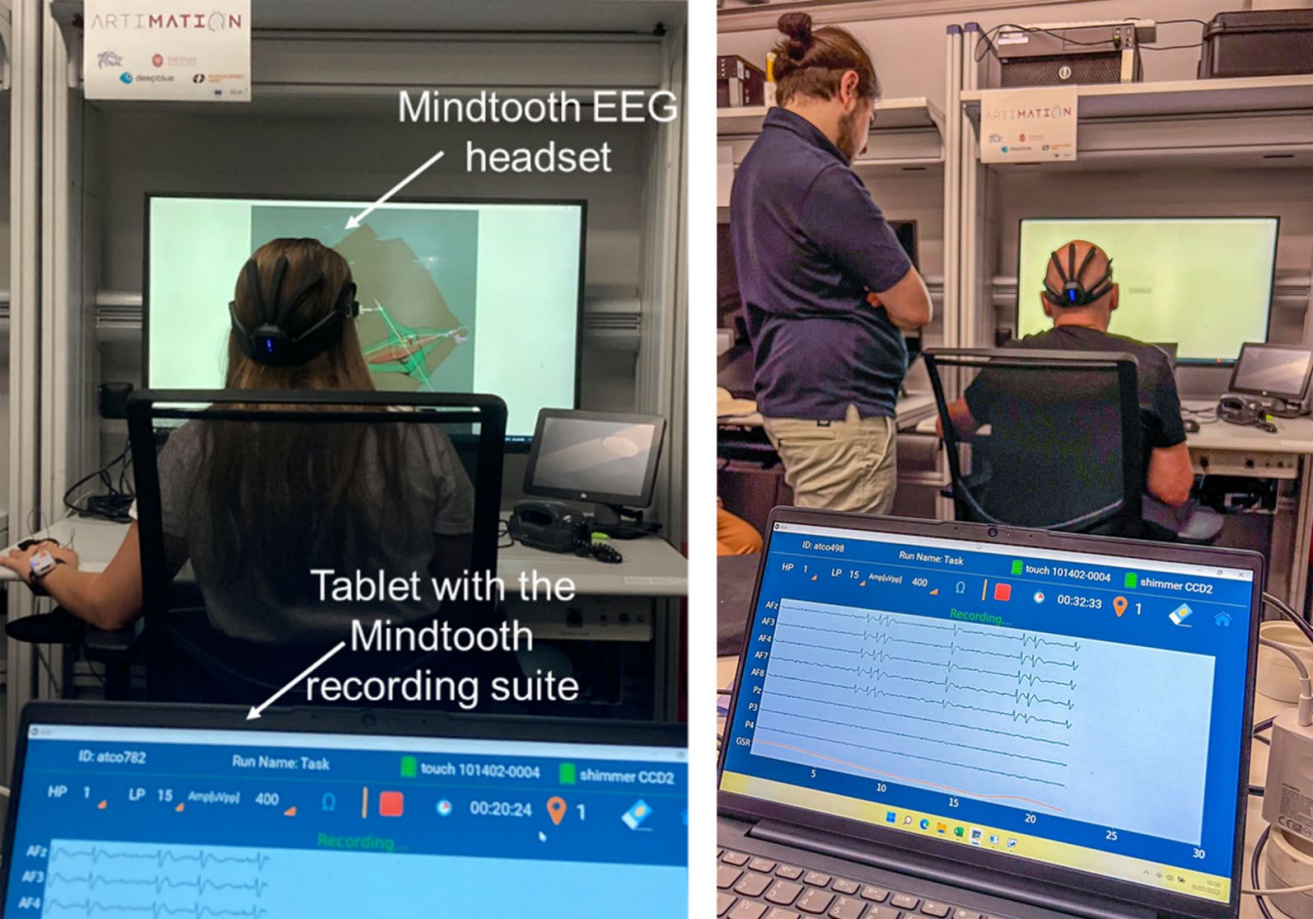
Validation setup for the neurophysiological measures

The participants were placed in the ACHIL En-Route control setting, ATC simulators facilities in ENAC, Toulouse, France. The control screen was either displaying the simulation, the solution and level of explanation, the drawing, or the survey (Fig. 4).

### Experimental design

**Fig. 5:**
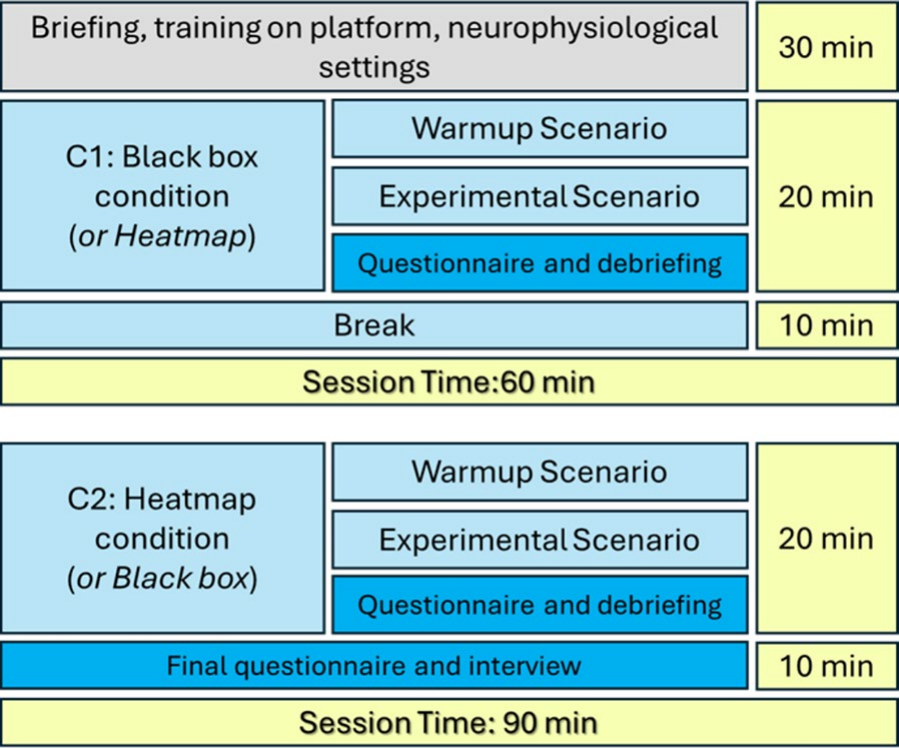
Experimental protocol and related timing

The experimental design (Fig. 5) aimed at testing primarily the different levels of explainability (BlackBox – No Explainability, and HeatMap – Explainability), while decreasing as much as possible any risk of bias, maximizing the quality of neurophysiological measures, and keeping the experiment short enough to not induce other confounding factors, such as mental fatigue. Before starting the experiment, each participant underwent an initial briefing and training session on the platform and the neurophysiological setting.

Every level of explanation was then tested twice. The first scenario was used as a warm-up (Warmup Scenario), while the following one (Experimental Scenario) was used to gather data for the analyses included in this paper, by randomizing the presentation of explainability. In particular, the researchers used a Latin square to randomize the order in which the level of explanation was presented to the participants to avoid any bias linked to the order of presentation, fatigue, or scenario.

At the end of each scenario, participants completed questionnaires and were briefly interviewed about their experience (see Questionnaire and debriefing section below for additional details). At the end of the experiment, the researchers conducted a qualitative interview with the participants to gather more insights into the presented XAI support. The experimental plan was designed to keep the total session under 1.5 h per participant.

### Collected data

#### Questionnaire measures

**Fig. 6:**
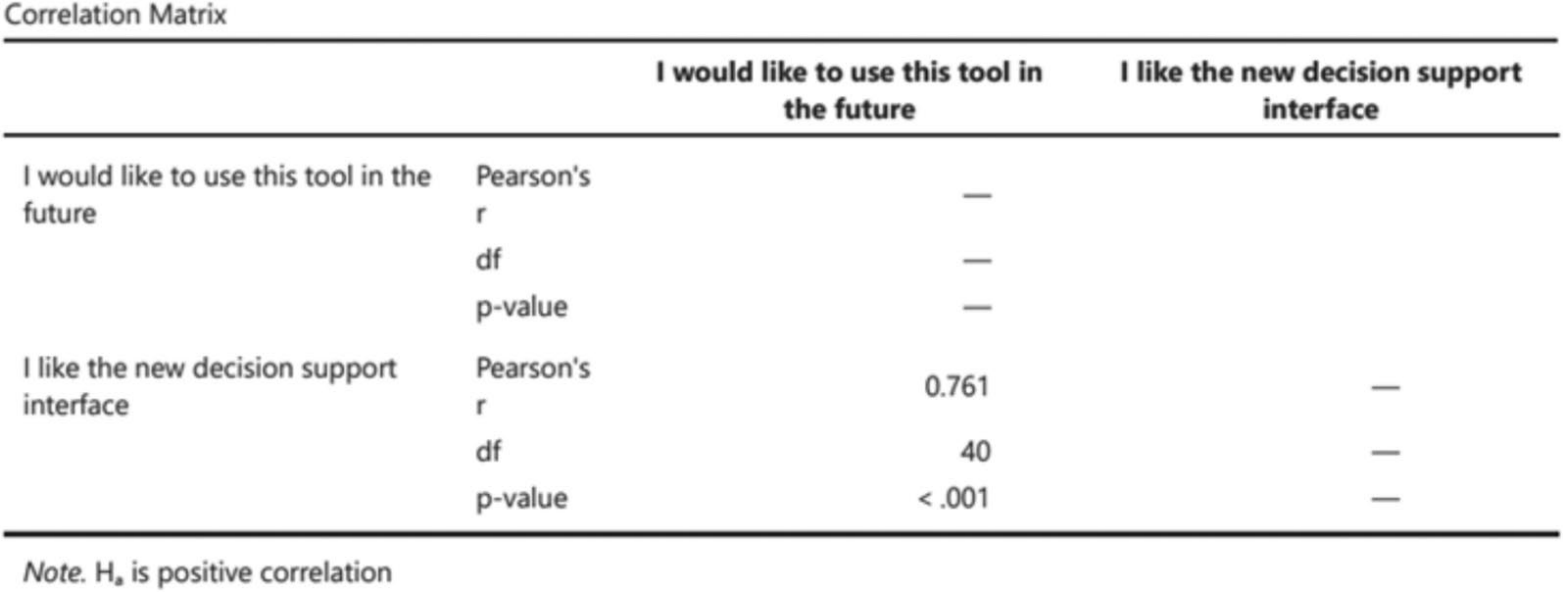
Correlation matrix for the willingness to use items (1)

**Fig. 7:**
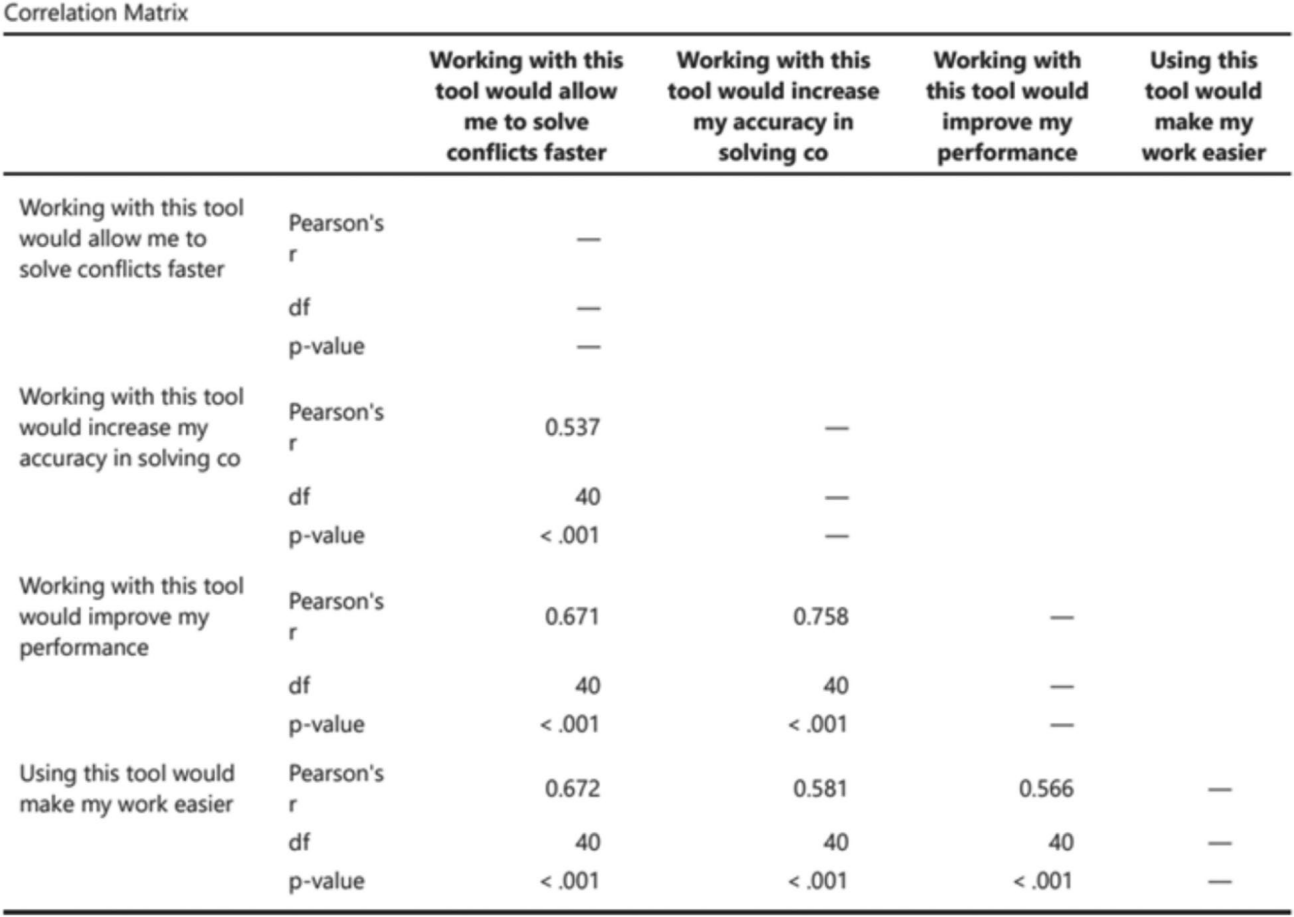
Correlation matrix for the willingness to use items (2)

At the end of each experimental scenario, participants were asked to complete a self-report, ad-hoc 3-question questionnaire assessing their agreement with the proposed solution and its comprehensibility, using a 5-point Likert Scale. At the end of each condition, the participants were also asked to fill in a self-report ad-hoc questionnaire to assess the willingness to use the XAI solution, the trust towards the support, the situational awareness gained through the usage of the support, the acceptability of the solution, and the general perceived impact on the work performance, on a 5-step Likert Scale (see Appendix A). Correlation analyses were conducted between related questionnaire items to verify whether they measured the same construct. Strong correlations justified aggregating items into composite indices (e.g., willingness-to-use and work performance), thereby improving reliability and simplifying statistical comparisons (Figs. 6 and 7).

#### Neurophysiological measures

Neurophysiological measures related to Mental workload and Acceptance have been obtained by a dedicated processing of the EEG signals. In the following, we provide a description of the mental states under consideration, with particular attention to the neurophysiological features associated with each of them and their related processing.

***Mental workload***

In recent decades, various definitions of mental workload have been proposed, indicating that it is a complex construct due to its multifaceted nature and the interplay of multiple cognitive aspects. The measurement of mental workload is a quantitative assessment of the mental activity resulting from performing a task. Several empirical investigations have indicated that performance declines at the extremes of the workload demand continuum - that is, when the event rate is excessively high or extremely low. For these reasons, the mental workload is an important and central construct in ergonomics and human factors research. It has already been shown in various contexts that the assessment of mental workload by EEG represents a reliable and objective measurement [43, 57].

This evidence showed that the brain’s electrical activities, fundamental for evaluating mental workload, are the theta and alpha EEG rhythms, located in the Pre-Frontal Cortex (PFC) and the Posterior Parietal Cortex (PPC) regions. The theta rhythm, especially over the PFC, presents a positive correlation, i.e., it increases when mental workload increases [58]. These results are also supported by the autonomic signals of heart rate (HR) and eye blink rate (EBR), by the performances’ trends and by the questionnaires for the evaluation of the perceived workload level, while the alpha rhythm, especially over the PPC, presents an inverse correlation, i.e. decreases [59]. In recent studies, it has also been demonstrated that machine learning techniques and specific brain features can be used to compute an EEG-based Workload Index, which is able to significantly discriminate workload demands during realistic tasks [60, 61].

***Acceptance (approach-withdrawal)***

Approach and withdrawal refer to the speed and ease with which a person adapts to new situations or changes. Some people adapt easily to new situations and tend to throw themselves into them, meet new people or try new things. Others, whose style is withdrawing, tend to need more time to warm up to new situations; they may hang back before they join in. That’s why this concept would be very useful to assess the acceptability and consequent motivation towards a certain stimulus. A large body of research on the relation between emotion and motivation has postulated the existence of two overarching motivational systems that organize behaviour. One system involves behaviour prompted by a possible desirable outcome, whereas the other involves behaviour prompted by a possible aversive outcome. A number of such models have been proposed, including Dickinson and Dearing’s, 1979 Aversive/Attractive systems, Gray’s Behavioural Activation/Behavioural Inhibition systems, and Lang et al.’s (1990) [62].

[62] proposed a similar model linked to research on frontal EEG asymmetry during emotional states. He posited that frontal asymmetry was not related to the valence of an emotional stimulus but rather to the motivational system that is engaged by that stimulus. He proposed that the left PFC is involved in a system facilitating approach behaviour to appetitive stimuli, whereas the right PFC is involved in a system facilitating withdrawal behaviour from aversive stimuli. It is so possible to derive an Approach-Withdrawal neurophysiological index by the processing of the EEG signal on the PFC. Such index has been already employed in a variety of fields and conditions supporting its consistency, like experimental conditions characterized by a low-, a neutral and a high-approach positive mindset [63], the study of personality traits in children, [64], but also public service announcement [65] and neuroaesthetics [66]. This type of index has a clear impact from an ergonomic perspective in operational fields, e.g., during the testing of new HMIs and technologies, to objectively assess the degree of acceptability of the operator in front of the new solution. In the following section it is reported the processing steps necessary to calculate the different neurophysiological measures from the EEG signal.

#### EEG data processing

The EEG signal was first band-pass filtered using a 5th-order Butterworth filter in the frequency range of 2-30 Hz. The blink artifacts have been identified and corrected by using the O-Clean algorithm [67]. EEG signals were then segmented into epochs of 1 s and if the EEG signal amplitude exceeds ±80 ($\mu$V) it was marked as artifact (threshold criterion, EEGlab [68]. From the artefact-free EEG, the Global Field Power (GFP) was calculated for each EEG frequency band of interest, on the Theta and Alpha bands. In this case, each band was defined accordingly to the Individual Alpha Frequency (IAF) value estimated for each participant [69]. Since the alpha peak is mainly prominent during rest conditions, the participants were asked to keep their eyes closed for a minute before starting the experiment. Such a condition was used to estimate the IAF value specifically for each participant. Consequently, the EEG bands are defined as:

1 $$\begin{aligned} & \Theta = (IAF - 6): (IAF - 2) Hz \end{aligned}$$

2 $$\begin{aligned} & \alpha = (IAF - 2): (IAF + 2) Hz \end{aligned}$$

For each mental state (Workload and Approach-Withdrawal), a subset of channels and bands of interest was chosen according to the literature [28, 66, 70–72].

The Workload neurometric was defined as:

3 $$\begin{aligned} WL = \frac{\Theta ~(Frontal~Channels)}{\alpha ~(Parietal~Channels)} \end{aligned}$$

The Acceptance neurometric was defined as:

4 $$\begin{aligned} AW = \alpha ~(Frontal~Dx) - \alpha ~(Frontal~Sx) \end{aligned}$$

## Results

Regarding subjective questionnaires, items with significant positive correlations were merged to simplify analyses and interpretation. These included combining the two “acceptability” items (“I would like to use this tool in the future" and "I like the new decision support interface”) into a “willingness to use” index ($r=0.761$, $p<0.001$), and merging the four “work performance” items (“Working with this tool would allow me to solve conflicts faster”, “Working with this tool would increase my accuracy in solving conflicts”, “Working with this tool would improve my performance”, “Using this tool would make my work easier”) into a composite index ($r=0.537$; $r=0.671$; $r=0.672$; $r=0.758$; $r=0.581$; $r=0.566$; $p<0.001$ for all the correlations) (Fig. 7).

Statistical comparisons, both for qualitative and quantitative results, have been performed using a repeated measures ANOVA (CI = 0.95), showing any within (Explainability) and between (Expertise) effects. In case of significant effects, the Duncan post-hoc test has been used to perform paired comparisons.

### Findings from semi-structured interviews

Qualitative data analysis has been conducted through semi-structured interviews in terms of Acceptance, Human Performance, Trust, Task and System Performance, XAI Applications in ATM, and Personalization of ML Algorithms. A short summary of the results is discussed below, and the details of these results can be found in [23, 27].

**Acceptance:** Students generally preferred the HeatMap (visual) condition for its clarity and appeal, though they also expressed interest in the BlackBox tool. Experts, however, were hesitant to accept AI-generated solutions due to concerns about time loss, lack of control, and being “out of the loop.” Experienced ATCOs noted that their conflict-resolution strategies-such as balancing penalties across multiple flights-were not fully captured by the algorithms. Experts ultimately favored the BlackBox solution, while students were more open to both HeatMap and BlackBox, highlighting generational and experience-based differences in acceptance and trust.

**Human performance:** Participants valued the BlackBox solution for its simplicity, minimal disruption, and familiarity with existing en-route tools. It provided single, clear solutions and useful trajectory details. Students appreciated the visual strengths of the HeatMap solution, such as its ability to highlight potential conflicts and incorporate factors like weather. However, experts cautioned that HeatMap could clutter displays and obscure critical information. Across groups, on-demand access to the tools was seen as most useful in high-workload or complex scenarios.

**Trust:** Trust in the AI tools was limited, largely due to insufficient training and a lack of transparency. Experts stressed the need for greater explainability to build confidence, while students were more willing to accept AI solutions on par with their own. Trust was viewed as essential before operational deployment, with training and debriefings identified as key avenues to foster it.

**Task and system performance:** The BlackBox solution was noted to facilitate faster conflict resolution, while HeatMap raised safety concerns due to clutter, cognitive tunneling, and excessive information. ATCOs worried about over-reliance on AI, potential skill degradation, and loss of vigilance over time. Mismatched AI proposals sometimes led controllers-especially students-to doubt their own solutions, risking delays or reduced situational awareness. AI support was deemed less useful for simple two-aircraft conflicts but potentially helpful in multi-aircraft scenarios.

**XAI Applications in ATM:** Higher explainability (HeatMap) was considered more valuable for training and planning tasks, where time is less critical, rather than for real-time operations. Explainability could help trainees understand algorithmic reasoning and build trust before transitioning to less visual, more operationally efficient tools. However, overreliance on AI in early training stages risks shaping novice strategies prematurely.

**Personalization of ML Algorithms:** Some ATCOs expressed interest in adaptive AI tools that learn individual control styles and strategies, aligning system recommendations with their decision-making preferences. Prior research confirms both common principles and individual variations in ATCO strategies, suggesting personalization could be a valuable future direction for AI support in conflict resolution.

### Questionnaire measures

#### Impact on work performance

**Fig. 8:**
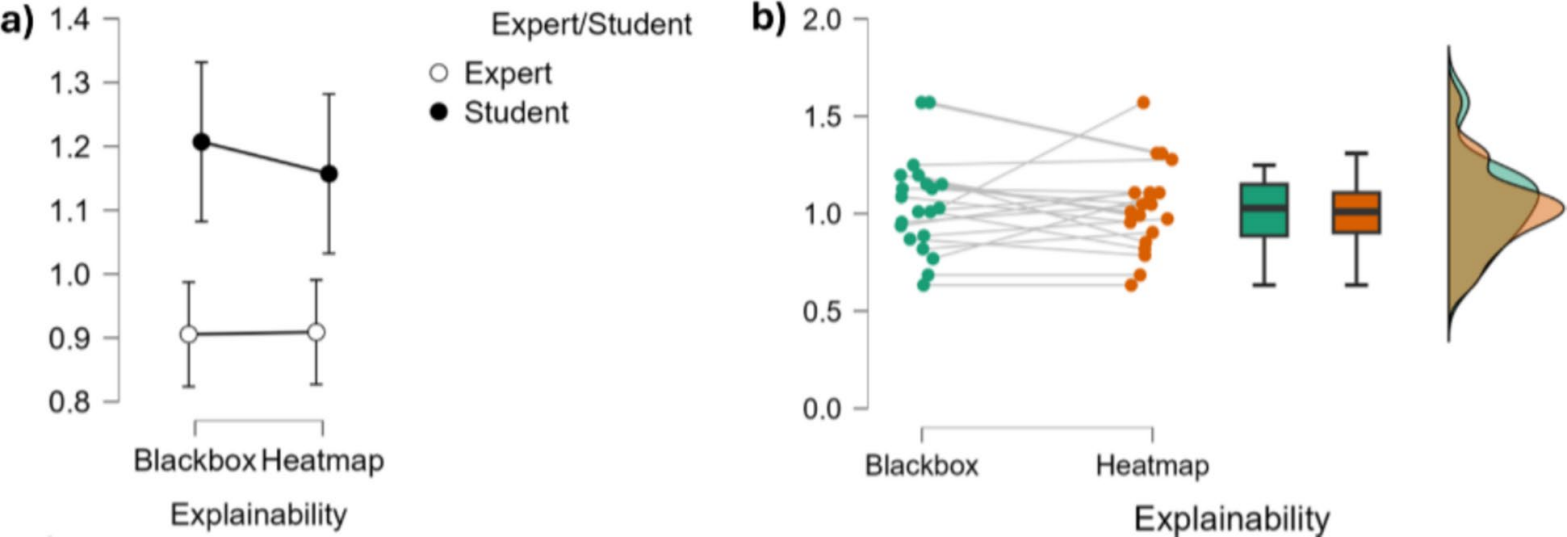
Top: Graphical representations of the impact of Explainability on work performances. **a** shows error plots regarding the level of expertise and Explainability, while **b** shows the individual points, the data distribution and the associated bar plots for explainability conditions

On the within-subjects effects level, the main effect of Explainability was not statistically significant regarding the impact on work performances ($F(1,19)=0.255,~p=0.620,~\eta ^2=0.003$), indicating that the presence or absence of explainability (i.e. HeatMap and BlackBox) did not significantly influence participants’ responses. Similarly, the interaction between Explainability and Expertise Level was not significant ($F(1,19)=0.337,~p=0.568,~\eta ^2=0.003$), suggesting that the influence of Explainability did not differ between experts and students (Fig. 8 and Table 2).

Regarding the between-subjects effects level, there was a significant main effect of Expertise Level on the impact on work performance ($F(1,19)=16.030,~p<.001,~\eta ^2=0.366$). This large effect indicates that experts and students differed significantly in their overall responses, according to their feelings about the impact on work performance, independent of the Explainability condition (Fig. 8 and Table 2).

**Table 2:** Statistical analyses tables displaying the impact of explainability on work performances for students and experts, as investigated through a repeated measures ANOVA (CI=0.95), both considering within-subjects effects (top) and between-subjects effects (bottom)

Source	Sum of squares	df	Mean square	F	*p*	$\eta ^2$
*Within-Subjects Effects*						
Explainability	0.006	1	0.006	0.255	.620	.003
Explainability $\times$ Expertise	0.007	1	0.007	0.337	.568	.003
Residuals	0.422	19	0.022			
*Between-subjects effects*						
Expertise	0.791	1	0.791	16.030	< .001	.366
Residuals	0.937	19	0.049			

Type III sum of squares reported. $\eta ^2$ = partial eta squared

#### Willingness to use

**Fig. 9:**
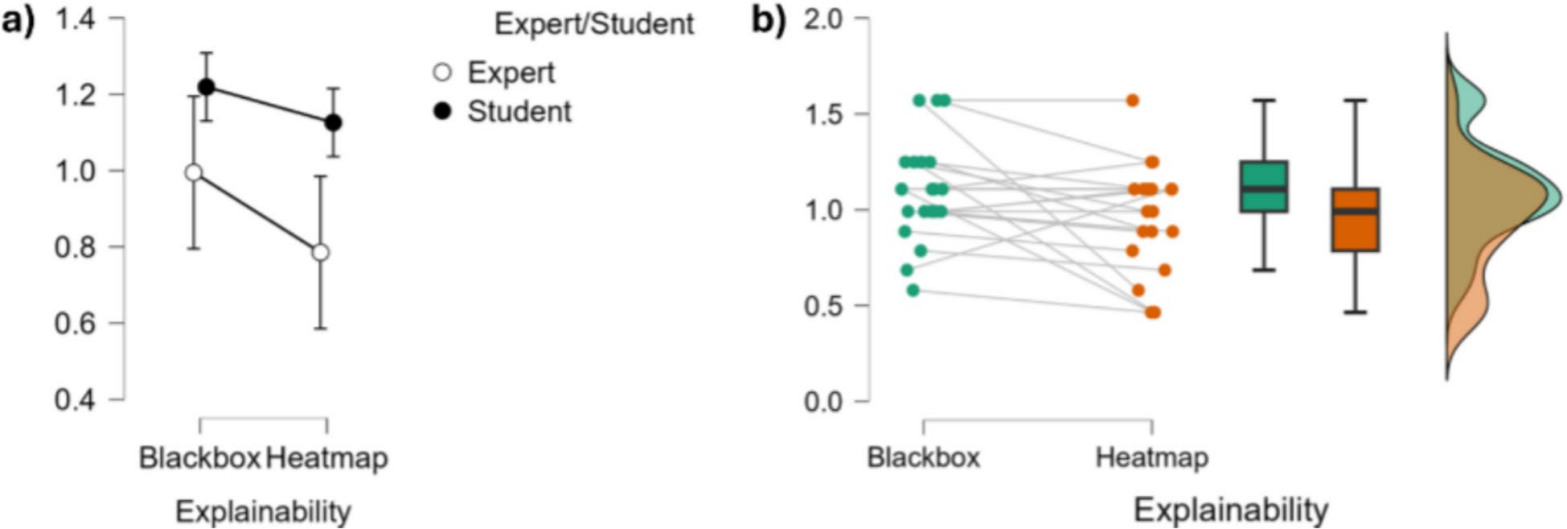
Top: graphical representations of the influence of explainability on willingness to use. **a** shows error plots regarding the level of expertise and explainability while **b** shows the individual points, the data distribution and the associated bar plots for explainability conditions

On the within-subjects effects level, there was a statistically significant main effect of Explainability on the willingness to use ($F(1,19)=4.453,~p=.048,~\eta ^2=0.072$), indicating that the presence of explainability (i.e., the HeatMap condition) significantly influenced participants’ responses across conditions (Fig. 9 and Table 3). However, the interaction between Explainability and Expertise Level was not statistically significant ($F(1,19)=0.657,~p=0.427,~\eta ^2=0.011$), suggesting that the effect of Explainability did not differ significantly between experts and students.

On the between-subjects effects level, there was a significant main effect of Expertise Level on the willingness to use ($F(1,19)=13.210,~p=0.002, \eta ^2=0.251$). This result indicates that experts and students differed significantly in their overall responses, regardless of the Explainability condition, with a large effect size (Fig. 9 and Table 3).

**Table 3:** Statistical analyses tables displaying the influence of explainability on willingness to use for students and experts, as investigated through a repeated measures ANOVA (CI=0.95), both considering within subjects effects (top) and between subjects effects (bottom)

Source	Sum of squares	df	Mean Square	F	*p*	$\eta ^2$
*Within-subjects effects*						
Explainability	0.240	1	0.240	4.453	.048	.072
Explainability $\times$ Expertise	0.035	1	0.035	0.657	.427	.011
Residuals	1.024	19	0.054			
*Between-subjects effects*						
Expertise	0.836	1	0.836	13.210	.002	.251
Residuals	1.203	19	0.063			

Type III sum of squares reported. $\eta ^2$ = partial eta squared

### Neurophysiological measures

#### Mental workload

**Fig. 10:**
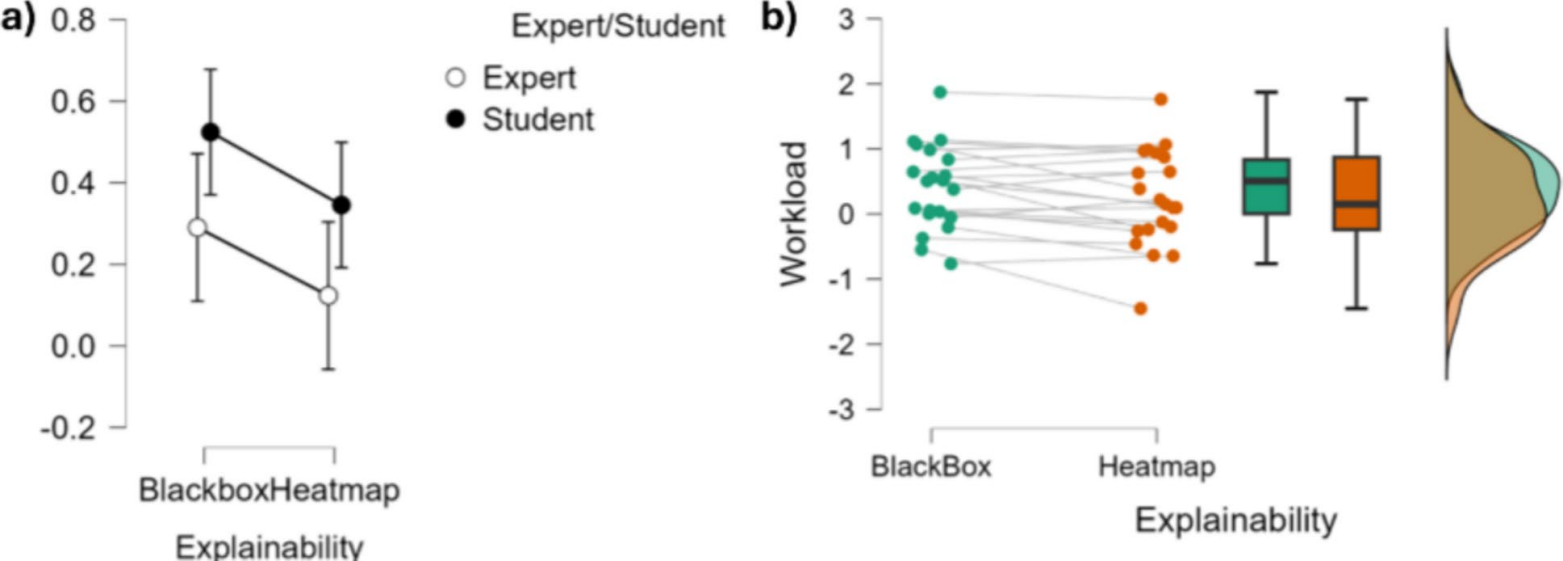
Top: graphical representations of the influence of Explainability on the measured mental workload. **a** shows error plots regarding the level of expertise and Explainability, while **b** shows the individual points, the data distribution and the associated bar plots for explainability conditions

At the within-subjects effects level, the analysis revealed a statistically significant main effect of Explainability ($F(1,19)=5.221, ~p = 0.034$), indicating that the presence of Explainability significantly affected participants’ mental workload (Fig. 10 and Table 4).

However, the interaction between Explainability and Expertise was not significant ($F(1,19)=0.006,~p=0.939$), suggesting that the effect of Explainability was similar across both expert and student groups. influenced participants

**Table 4:** Bottom: statistical analyses tables displaying the influence of explainability on the measured mental workload for students and experts, as investigated through a repeated measures ANOVA (CI=0.95), both considering the within-subjects effects (top) and between-subjects effects (bottom)

Source	Sum of squares	df	Mean square	F	*p*	$\eta ^2$
*Within-subjects effects*						
Explainability	0.313	1	0.313	5.221	0.034	0.016
Explainability $\times$ Expertise	3.580$\times$10^− 4^	1	3.580$\times$10^− 4^	0.006	0.939	1.822$\times$10^− 5^
Residuals	1.139	19	0.060			
*Between-subjects effects*						
Expertise	0.544	1	0.544	0.586	0.454	0.028
Residuals	17.652	19	0.929			

Type III sum of squares reported. $\eta ^2$ = partial eta squared. Sphericity corrections not applicable for factors with two levels

Regarding the between-subjects effects level, no significant main effect of Expertise Level on measured mental workload was found ($F(1,19)=0.586,~p=0.454$), indicating that overall differences between experts and students were not statistically significant, regardless of the Explainability condition (Fig. 10 and Table 4).

#### Acceptance (approach-withdrawal)

**Fig. 11:**
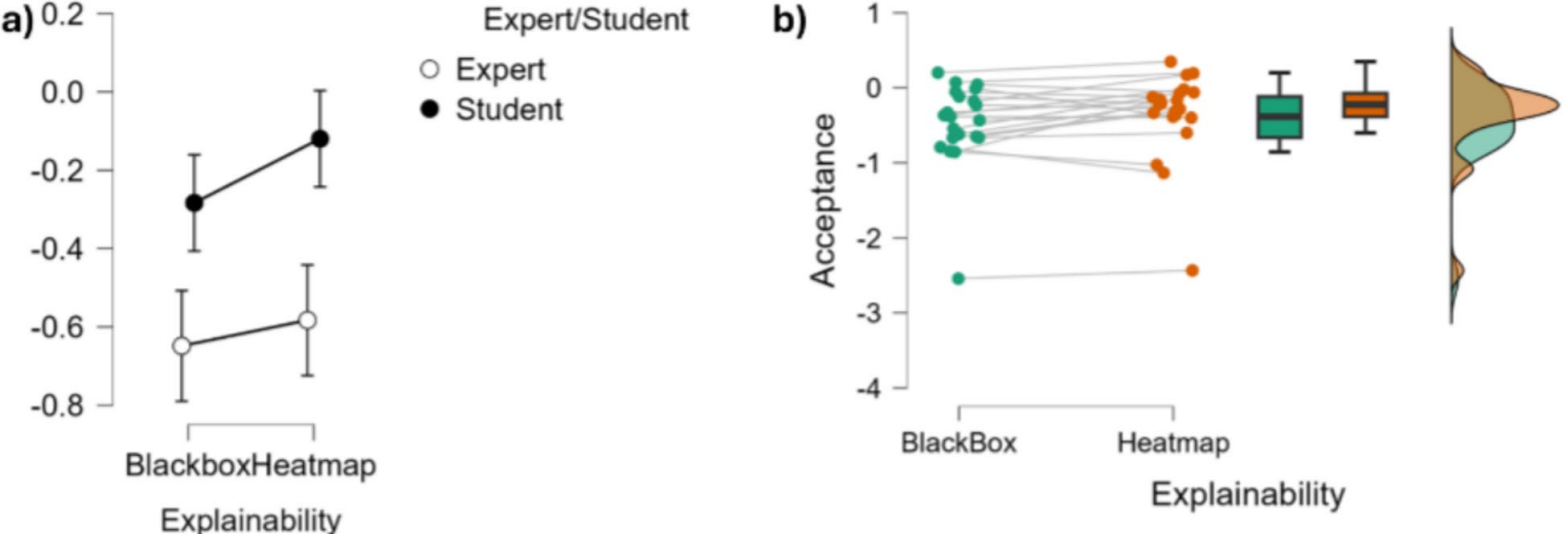
Top: Graphical representations of the influence of explainability on the measured acceptance neurometric. **a** shows error plots related to the levels of Expertise and Explainability while **b** shows the individual points, data distribution and associated bar plots for explainability conditions

On the within-subjects effects level, the main effect of Explainability on the measured acceptance approached statistical significance ($F(1,19)=3.696,~p=0.070,~\eta ^2=0.163$), suggesting a potential trend toward Explainability affecting participants’ acceptance while using the system. The interaction between Explainability and Expertise was not significant ($F(1,19)=0.675,~p=0.421,\eta ^2=0.034$), indicating that the effect of Explainability was similar for both experts and students (Fig. 11 and Table 5).

Concerning the between-subjects effects level, the main effect of Expertise Level was also found not statistically significant ($F(1,19)=3.116,~p=0.094,~\eta ^2=0.141$), though it showed a trend toward significance with a moderate effect size. In this regard, a Duncan post hoc test revealed that students exhibited an increase in acceptance (trend, $p=0.067$ over the HeatMap condition) (Fig. 11 and Table 5).

**Table 5:** Statistical analysis tables displaying the influence of explainability on the measured acceptance neurometric for students and experts, as investigated through a repeated measures ANOVA (CI = 0.95), both considering within-subjects effects (top) and between-subjects effects (bottom)

Source	Sum of squares	df	Mean Square	F	*p*	$\eta ^2$
*Within-subjects effects*						
Explainability	0.138	1	0.138	3.696	.070	.163
Explainability $\times$ Expertise	0.025	1	0.025	0.675	.421	.034
Residuals	0.708	19	0.037			
*Between-subjects effects*						
Expertise	1.798	1	1.798	3.116	.094	.141
Residuals	10.967	19	0.577			

Note. Type III sum of squares reported. $\eta ^2$ = partial eta squared

## Discussion

The present study set out to investigate how visual explanations, specifically HeatMap-based XAI, influence cognitive workload, user acceptance, and intention to adopt AI decision-support tools in the context of ATC. Recognizing the critical need for explainability in safety-critical domains, the experiment compared a black-box AI condition with a HeatMap-explanation condition across two distinct user groups: expert ATCO instructors and student controllers. By integrating objective neurometrics derived from EEG with subjective questionnaire data, this research provided a comprehensive assessment of both implicit and explicit responses to AI explanations. The study aimed not only to evaluate the direct effects of explainability on human factors but also to explore how the level of user expertise moderates these effects. In doing so, it addressed key gaps identified in the literature regarding the operational impact of XAI in ATM and contributed empirical evidence to support user-centered design principles in the development of trustworthy AI systems [15, 73, 74].

### Summary of key findings

This study examined the influence of XAI, specifically HeatMap-based visualizations, on cognitive workload, user acceptance, and willingness to adopt AI decision-support tools within ATC. Recognizing the critical need for transparency and comprehensibility in safety-critical systems, the study assessed both explicit (subjective questionnaires) and implicit (EEG-based neurometrics) user responses, comparing experienced ATCOs with student controllers.

Subjective questionnaire data revealed no significant influence of explainability on participants’ perceived impact on work performance, indicating that their explicit evaluations were unaffected by the presence or absence of visual explanations. However, a significant main effect of expertise emerged, revealing that students consistently reported a higher perceived impact of AI support on their performance than expert ATCOs. This suggests that novices, who are potentially less influenced by established routines or biases, may perceive AI assistance as more influential in their work, highlighting the important role of user experience and generational factors in shaping perceptions toward AI integration.

Furthermore, participants expressed significantly greater willingness to use the AI system when provided with explainability (HeatMap condition), compared to the black-box condition. This finding highlights the crucial role of visual explanations in promoting user acceptance of AI-based support tools. Additionally, the significant difference between expert and student ATCOs in their overall willingness to adopt the AI tools suggests that younger, less experienced controllers may be more receptive to integrating novel technologies into their workflows, further emphasizing expertise as a key moderator in technology acceptance.

Neurometric measures, derived from EEG recordings, demonstrated a significant reduction in cognitive workload under the HeatMap-based explainability condition compared to the black-box condition, independent of user expertise. This critical finding provides objective evidence supporting the hypothesis that visual explanations effectively reduce cognitive demands by clarifying AI-generated recommendations, enabling users to allocate their cognitive resources more efficiently.

Finally, implicit measures of acceptance (Approach-Withdrawal metrics) revealed a trend towards higher acceptance levels in the presence of explainability, although this effect did not reach conventional levels of statistical significance. No significant differences were observed between the expert and student groups in this measure; however, post hoc analyses revealed a marginal trend for increased implicit acceptance among students in the HeatMap condition. This suggests that visual explanations may positively influence user perceptions at an implicit level, particularly among less experienced users, warranting further exploration.

Taken together, these results emphasize that visual explanations, such as HeatMap, positively impact user acceptance and cognitive efficiency when interacting with AI tools in ATC contexts. However, explicit perceptions of AI’s impact on work performance appear more closely related to individual expertise and generational differences than to the presence of explainability alone. These findings provide a nuanced view of how explainability interacts with user characteristics and cognitive factors, preparing the ground for deeper discussion on designing AI systems that effectively align technical performance with human-centered requirements in safety-critical domains.

### Theoretical and practical implications

This study found that expertise significantly influences how users perceive the impact of AI on work performance, and less experienced operators (students) reported a greater perceived impact from AI systems compared to experienced ATCOs. These results are consistent with a previous study in which the domain-specific expertise significantly impacted how users trust and engage with explainable AI systems. More experienced users tend to have lower initial trust in AI and demand clearer explanations, whereas less experienced users are more influenced by the perceived intelligence of the system itself [75]. These findings suggest that AI integration strategies in ATM should consider the users’ expertise and age [36, 76]. Training programs for AI systems could be tailored to address the specific needs and biases of different user groups, ensuring that both novice and experienced operators can effectively adopt and utilize AI technologies [77]. Additionally, this underscores the importance of designing AI systems that can accommodate diverse user profiles, potentially leading to more universal adoption and improved operational performance across varying levels of expertise.

Additionally, both user experience and the type of AI explanation affect willingness to use AI systems, with less experienced operators showing a higher willingness to use AI, likely due to fewer biases towards traditional methods. The preference for BlackBox conditions among some experienced ATCOs highlights the complexity of trust in AI systems and reinforces the previous literature [78]. Practically, this indicates the need for a flexible approach in AI design, offering both detailed explanations and more opaque options depending on the user’s preference and trust level. Theoretically, it suggests a nuanced relationship between transparency and trust, where more information isn’t always better. Providing excessive or complex AI explanations can lead to cognitive overload and reduce effective trust. Adjusting the amount and type of explanation based on task complexity improves both trust and performance [79]. Consequently, developing adaptive AI systems that can adjust the level of explanation based on user feedback and context could thus enhance acceptance, usability and trust of the user [73, 80].

The study also revealed that explainability significantly affects workload, independent of user expertise. Both students and experts ATCOs experienced reduced workload with the HeatMap condition compared to the BlackBox condition. This indicates that clear, visual AI explanations can alleviate cognitive load by allowing users to focus on essential task aspects without delving into the AI’s internal workings [81–83]. Thus, XAI can enhance efficiency in conflict resolution tasks by reducing the cognitive demands on users. The reduction in cognitive load through explainable AI suggests that visual tools, such as HeatMap, could be broadly implemented to enhance operational efficiency in ATM. Practically, incorporating these tools could lead to fewer errors and quicker decision-making. Theoretically, this supports the cognitive load theory [84], indicating that well-designed XAI systems can serve as cognitive aids, improving user performance by simplifying complex information processing [85, 86]. This also implies that future AI development should focus on creating intuitive, easily interpretable visual aids to support high-stakes decision-making.

It is also important to recognize that the explanatory needs of controllers are not homogeneous across ATC roles. Tower, approach, and en-route controllers operate under markedly different temporal constraints and cognitive demands, which shapes how explanations should be designed and presented. Tower control requires immediate, tactical interventions in highly dynamic environments; in this setting, explanations need to be extremely concise and directly actionable, as any additional cognitive burden could compromise safety. Approach control combines both tactical sequencing and broader sector coordination, suggesting that explanations may need to balance brevity with contextual justification [87]. En-route control, by contrast, involves longer time horizons and broader situational management, making more elaborative and exploratory explanations [88], such as the HeatMap-based visualization tested here, particularly relevant. Because most experts in our sample had extensive en-route experience, our findings are most directly interpretable in this context. Nevertheless, future research should investigate how different explanation modalities may need to be adapted to role-specific operational environments, ensuring that XAI interfaces are responsive to the distinct cognitive ecologies of tower, approach, and en-route operations.

Furthermore, the type of AI explanation and user experience both influence implicit user reactions, with the HeatMap condition eliciting more positive implicit responses compared to the BlackBox condition, suggesting that visual explanations help users anchor their decision-making process. The positive response to HeatMap explanations suggests that visual anchors are effective tools for enhancing user acceptance and trust in AI systems [89–91]. Practically, this means that incorporating visual elements that provide clear, actionable insights can improve user interaction with AI. Theoretically, this reinforces the importance of anchoring heuristics in decision-making processes, suggesting that XAI designs should incorporate these principles to facilitate more effective cognitive processing and user satisfaction. Future research should investigate other forms of visual aids that can serve similar anchoring functions across various contexts and user groups.

An additional theoretical lens relevant to these findings is the concept of we-agency, which refers to the experience of feeling a sense of control over actions performed by a partner during a collaborative task [92–94]. In the context of human–machine interactions, this sense of we-agency is often attenuated as machine-generated actions typically fall outside the human motor repertoire and are therefore less likely to be integrated into the individuals’ sensorimotor predictions [95, 96]. As a result, building a sense of shared control with AI systems remains challenging. In our study, however, the positive effects of HeatMap-based XAI explanations, including reduced cognitive workload and more favorable implicit responses, suggest that visual cues may serve as cognitive scaffolds that help users simulate shared understanding or even rudimentary forms of co-agency. This effect appeared more pronounced among less experienced users, who reported a greater impact and a higher willingness to adopt the system, possibly due to greater cognitive flexibility or less anchorage to established procedural routines than experienced users [97–99]. Conversely, some experienced ATCOs preferred black-box AI, potentially reflecting a reluctance to cognitively engage with systems that disrupt established workflows [100, 101]. Taken together, these findings highlight the need for neuroadaptive or expertise-sensitive XAI systems that can dynamically tailor explanations to the user’s mental model and cognitive needs. By promoting internalization of machine behaviour and aligning explanatory strategies with the user’s expertise, such systems could reinforce effective we-agency, enhance trust calibration, and improve coordination fluency in human-AI teams within safety-critical environments [22, 92]. Future research should explore other forms of visual aids that can serve similar anchoring functions across various contexts and user groups.

Finally, in their comprehensive survey addressing the opacity of complex AI systems, [20] proposed a unified, user-centered definition of explainability alongside structured taxonomies for general machine learning models and deep learning approaches, highlighting critical challenges such as the interpretability-performance trade-off, the lack of standardized evaluation metrics, and the need for context-aware, user-adapted explanations. Similarly, [21] emphasized an urgent shift toward user-centric XAI systems in ATM, stressing the necessity of aligning AI outputs closely with the operational needs and cognitive models of ATCOs. Addressing these critical gaps, the current research significantly contributes to the XAI domain by empirically elucidating how visual explanations, specifically HeatMap-based XAI, influence cognitive workload, user acceptance, and intention to adopt AI decision-support tools within ATC. By differentiating user responses based on expertise, this work offers nuanced insights that inform targeted strategies for AI training and deployment tailored to diverse user groups. Moreover, by combining objective neurophysiological metrics (EEG) with subjective assessments, this study advances methodological rigor in human-AI interaction evaluations, directly responding to recent calls for multimodal approaches in XAI research [19, 25, 102], thereby laying practical foundations for enhancing operational efficiency, reducing cognitive fatigue, and supporting user trust in AI-supported ATC environments.

## Limitations

While our findings provide promising evidence, the limited sample size constrains generalizability. Results should be considered exploratory, warranting replication with larger and more representative cohorts. Indeed, due to their professional duties and operational responsibilities, ATCOs are notoriously challenging to recruit for experimental studies. Recruitment processes typically must be initiated very early, often requiring extensive coordination with operational management. This limitation underscores the need for caution in broadly interpreting the findings and suggests that future studies should strive to secure larger and more representative samples to enhance statistical power and generalizability.

Furthermore, the exploratory and preliminary results discussed in this paper are specific to the ATM domain and may not be directly generalizable to other safety-critical domains. Each domain has unique operational requirements, user profiles, and varying levels of AI integration, all of which significantly impact the transferability of results. Even within aviation, the applicability of these findings can vary considerably. For instance, the operational requirements and explanatory needs differ substantially between en-route ATCOs, who manage long-term traffic flow over expansive airspace, and tower or approach sector ATCOs, who handle immediate, detailed tasks such as take-offs, landings, ground movements and sequencing. Given that most of our expert participants had extensive en-route experience (with some having additional tower/approach backgrounds), the results are most directly interpretable for the en-route context. However, we explicitly acknowledge that different explanation modalities may be required for tower and approach control. For example, in high-density tower operations, simpler visual cues (e.g., colour-coded alerts or minimal graphical overlays) may be preferable to full HeatMap. Such variability implies that a one-size-fits-all approach to XAI may not be effective, highlighting the necessity for tailored explanations that address the distinct demands of each role according to each task [73, 103, 104]. Indeed, recent studies further emphasize that domain-specific expertise significantly affects trust in AI systems, suggesting explanations might be particularly critical for highly experienced users [75].

Another limitation of the present work lies in the categorization of participants into only two groups: “experts” and “students.” While this dichotomy allowed for clear statistical contrasts given the limited sample size, it oversimplifies the real spectrum of expertise in ATC. In practice, expertise is a continuum shaped not only by years of experience but also by sector-specific exposure (tower, approach, en-route), the phase of professional training, and generational familiarity with novel technologies. Intermediate groups, such as recently licensed controllers in their first years of duty, or controllers who operate in specific sectors (e.g., only approach control), may show distinct patterns of trust, workload management, and reliance on AI explanations compared to both novices and highly experienced instructors [105]. Indeed, prior research suggests that early-career professionals often rely more heavily on external aids while developing internalized strategies, whereas very experienced operators may resist automation that conflicts with established routines [106]. Capturing these nuances would provide a richer understanding of how explanatory interfaces should adapt across the full career trajectory. Future research should thus include such intermediate categories to capture more nuanced insights into how expertise modulates workload, trust, and acceptance of AI explanations across the career trajectory of controllers.

A further limitation of the present study is that only a HeatMap-based visual explanation was tested against a black-box condition. While this choice reflects the strong visual orientation of ATC work and the need for explanations that integrate directly into radar-like displays, it does not allow conclusions about other explanation modalities. Prior research in visual analytics and cognitive ergonomics suggests that textual explanations can monopolize attention and divert cognitive resources away from monitoring tasks [107], making them less suitable for real-time operational settings. By contrast, hybrid approaches, such as combining minimal text with schematic visuals, may offer richer support in training or planning scenarios, where time constraints are less acute. Future studies should therefore systematically compare different explanation modalities, including visual, textual, and hybrid formats, across ATC roles and contexts. Such comparative research would help identify when and for whom each modality is most beneficial, ultimately informing the design of flexible XAI systems that adapt explanation styles to both task demands and user preferences.

Finally, from a methodological perspective, several constraints warrant explicit acknowledgment. Electroencephalography, while providing objective neurophysiological insights, comes with inherent methodological limitations, including susceptibility to noise, potential confounding factors such as fatigue or stress, and challenges related to data interpretation due to the complexity of cognitive workload constructs. Additionally, the subjective assessment of user perceptions employed in this study was limited to only five questionnaire items, which may have restricted the breadth and depth of the captured user experiences. Another limitation is the absence of direct behavioral performance metrics such as conflict-resolution speed, accuracy, and error rates. While EEG-based neurometrics and self-reports provided complementary insights into workload and acceptance, the lack of behavioral data prevents us from confirming whether these cognitive and perceptual benefits translate into measurable improvements in operational performance. Future studies should therefore integrate behavioral logging alongside neurophysiological and subjective assessments to provide a more comprehensive evaluation of human–AI interactions. Moreover, although the present study was conducted in ENAC’s high-fidelity ACHIL en-route simulator, which offers a realistic approximation of operational conditions, simulators cannot fully reproduce the unpredictability and pressures of live operations. Thus, the transferability of these findings to real-world ATC, particularly in tower and approach sectors, remains to be established, and further research in live or shadow-mode operational environments is required.

## Conclusions

This study provides critical insights into the role of visual explanations, specifically HeatMap-based XAI, in shaping cognitive workload, user acceptance, and adoption intentions within ATC environments. By addressing existing gaps in the literature concerning the empirical evaluation of XAI systems, this work demonstrated that visual explanations significantly alleviate cognitive load and enhance user acceptance (through willingness to use), underscoring the practical value of transparent, interpretable AI systems in safety-critical domains. Our findings emphasize the nuanced role of user expertise, highlighting that while visual explanations universally reduce cognitive workload and foster positive adoption intentions, explicit perceptions of AI impact are notably influenced by user experience. Less experienced users exhibited greater receptivity to AI-driven tools, suggesting that expertise and generational factors significantly shape the integration and perceived utility of novel technologies. Furthermore, this research underscores the methodological importance of employing multimodal evaluation frameworks, which integrate both objective neurophysiological measures (EEG) and subjective user feedback, thereby providing a more comprehensive understanding of human-AI interactions. Overall, this study contributes foundational knowledge for developing cognitively aligned, user-centric AI tools that effectively balance technological innovation with human needs, ultimately enhancing operational efficiency, reducing cognitive strain, and boosting trust in AI-supported ATC systems.

## Figures and Tables

**Fig. 12:**
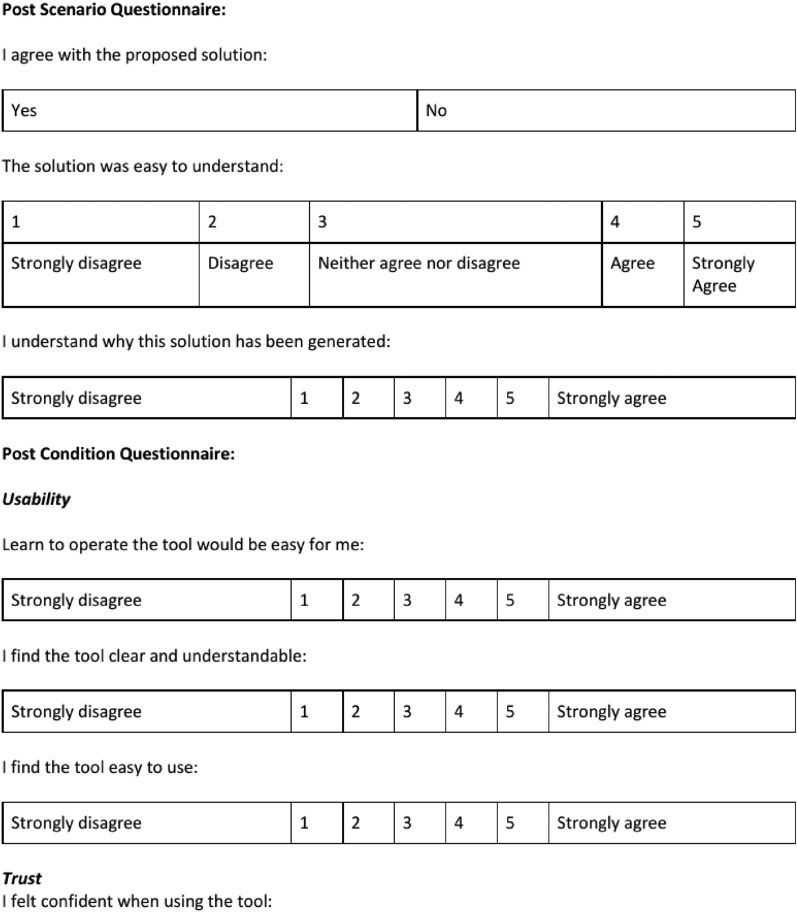
Questionnaire used during the experimentation (1)

**Fig. 13:**
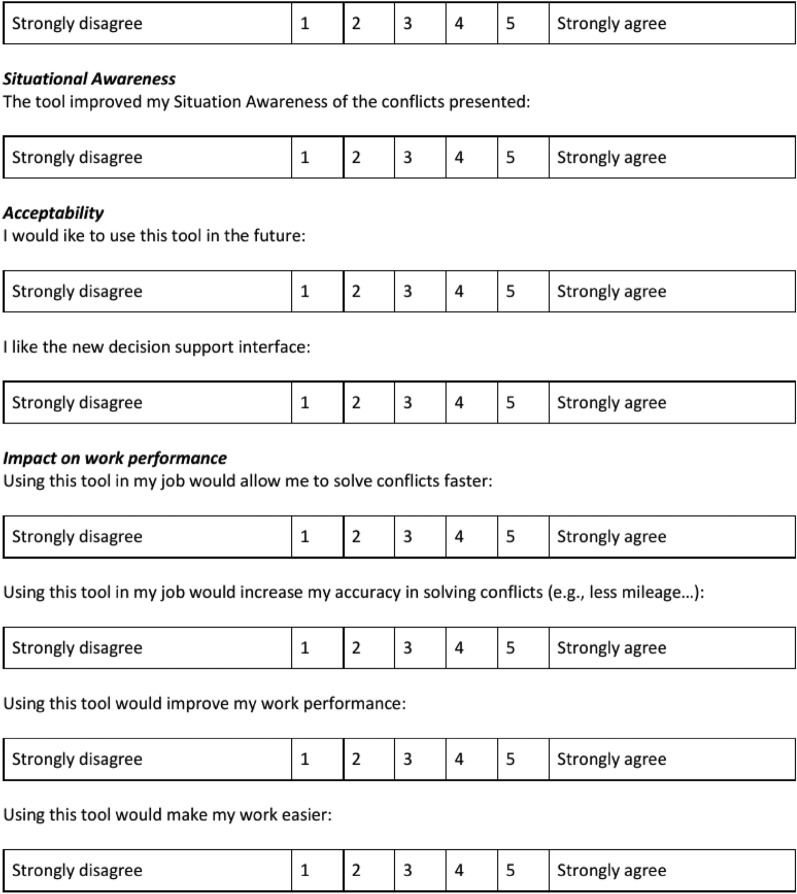
Questionnaire used during the experimentation (2)

## Data Availability

The datasets generated and/or analysed during the current study are available in the ARTIMATION H2020 Project ZENODO repository: https://zenodo.org/records/7437564; https://zenodo.org/records/7437969; https://zenodo.org/records/7437777.

## Appendix A Questionnaires

In this appendix are presented the questionnaires used during the experimentation with air traffic controllers. Each condition (BlackBox or HeatMap) was followed by two questionnaires: a 3-item solution agreement/comprehension questionnaire (assessing agreement with the proposed solution, perceived comprehensibility, and confidence in the solution); and a multi-item willingness-to-use/trust/acceptance questionnaire (assessing willingness to use the system in the future, trust in the system, situational awareness, acceptance, and perceived impact on work performance).

See Figs. 12 and 13

## Footnotes

1 www.mindtooth-eeg.com.
